# Anti‐β‐sheet conformational monoclonals safely reduced pathology in TgSwDI/apoE2, 3 and 4 AD mouse models

**DOI:** 10.1002/alz70859_102000

**Published:** 2025-12-25

**Authors:** Fernando Goni, Ding Wang, Jianina Suazo, Jasmine Lin, Jasmine Okeagu, Begonia Gamallo‐Lana, Adam Mar, Thomas Wisniewski

**Affiliations:** ^1^ NYU Grossman School of Medicine, New York, NY USA; ^2^ New York University School of Medicine, New York, NY USA; ^3^ New York University Grossman School of Medicine, New York, NY USA

## Abstract

**Background:**

We have demonstrated an IgM monoclonal antibody (GW‐23B7) which targets specific β‐sheet secondary structure present in all AD pathologic oligomers (AβComAb) produces significant cognitive rescue linked to lowering Aβ oligomers in 3xTg and TgSwDI mouse models, without inducing inflammation or microhemorrhages. We have now tested the efficacy and safety of two AβComAbs, GW‐23B7 and WG‐3D7 in the cerebral amyloid angiopathy (CAA) TgSwDI model knockin for human apoE2, 3 or 4.

**Method:**

For TgSwDI, TgSwDI/apoE2, TgSwDI/apoE3 and TgSwDI/apoE4 mice; three groups of 11 months AD animals were used for the preclinical trial. Each group was inoculated i.p. weekly for two months with 100 µg/animal of either GW‐23B7 or WG‐3D7 in sterile saline or sterile saline vehicle Control alone. The animals then underwent locomotor and behavioral cognitive testing followed by the brains being harvested for immunohistochemical and biochemical analysis.

**Result:**

The intact IgMs GW‐23B7 and WG‐3D7 interacted with the brain vasculature and penetrated the BBB in the treated groups without inducing microhemorrhages as determined by Prussian Blue staining. Moreover, the treated groups showed significant cognitive rescue compared to the controls on the Barnes Maze, which correlated with a significant decrease of soluble aggregated Aβ in specific ELISA tests, and a significant decrease of vascular and parenchymal Aβ pathology, with some reduction of activated glia shown by Iba1, GFAP and CD‐45 immunohistochemistry. Small differences were found between the effects of the 2 AβComAbs.

**Conclusion:**

We have shown that peripherally infused AβComAbs, IgMs GW‐23B7 and WG‐3D7 reduce, independently of all the human ApoE allotypes, soluble oligomeric and deposited vascular and parenchymal Aβ pathology by an Aβ‐sequence independent mechanism avoiding ADCC induced microhemorrhages or inflammation. Our preclinical data testing the therapeutic efficacy of IgMs GW‐23B7 and WG‐3D7 in multiple AD/apoE models suggests that they could be safely translated to human AD clinical trials with a minimal possibility of ARIA or autoimmune toxicity complications.

